# Fecal microbiota transplantation inhibited neuroinflammation of traumatic brain injury in mice via regulating the gut–brain axis

**DOI:** 10.3389/fcimb.2023.1254610

**Published:** 2023-09-07

**Authors:** Xuezhen Hu, Hangqi Jin, Shushu Yuan, Tao Ye, Zhibo Chen, Yu Kong, Jiaming Liu, Kaihong Xu, Jing Sun

**Affiliations:** ^1^ Department of Emergency Medicine, The Second Affiliated Hospital and Yuying Children’s Hospital of Wenzhou Medical University, Wenzhou, China; ^2^ Department of Preventive Medicine, School of Public Health and Management, Wenzhou Medical University, Wenzhou, China; ^3^ Department of Geriatrics, The Second Affiliated Hospital and Yuying Children’s Hospital of Wenzhou Medical University, Wenzhou, China; ^4^ Department of Hematology, Ningbo First Hospital, Ningbo, China

**Keywords:** traumatic brain injury, fecal microbiota transplant, neuroinflammation, gut microbiota, gut–brain axis, Treg cell

## Abstract

**Introduction:**

Recent studies have highlighted the vital role of gut microbiota in traumatic brain injury (TBI). Fecal microbiota transplantation (FMT) is an effective means of regulating the microbiota–gut–brain axis, while the beneficial effect and potential mechanisms of FMT against TBI remain unclear. Here, we elucidated the anti-neuroinflammatory effect and possible mechanism of FMT against TBI in mice via regulating the microbiota–gut–brain axis.

**Methods:**

The TBI mouse model was established by heavy object falling impact and then treated with FMT. The neurological deficits, neuropathological change, synaptic damage, microglia activation, and neuroinflammatory cytokine production were assessed, and the intestinal pathological change and gut microbiota composition were also evaluated. Moreover, the population of Treg cells in the spleen was measured.

**Results:**

Our results showed that FMT treatment significantly alleviated neurological deficits and neuropathological changes and improved synaptic damage by increasing the levels of the synaptic plasticity-related protein such as postsynaptic density protein 95 (PSD-95) and synapsin I in the TBI mice model. Moreover, FMT could inhibit the activation of microglia and reduce the production of the inflammatory cytokine TNF-α, alleviating the inflammatory response of TBI mice. Meanwhile, FMT treatment could attenuate intestinal histopathologic changes and gut microbiota dysbiosis and increase the Treg cell population in TBI mice.

**Conclusion:**

These findings elucidated that FMT treatment effectively suppressed the TBI-induced neuroinflammation via regulating the gut microbiota–gut–brain axis, and its mechanism was involved in the regulation of peripheral immune cells, which implied a novel strategy against TBI.

## 1 Introduction

Traumatic brain injury (TBI) is one of the leading causes of incidence rate and mortality worldwide, which has brought a huge burden to society, the economy, and medical care (Jiang et al., 2019). Mechanical brain injury initiates inflammatory and neurovascular changes in the brain, which can lead to neuronal injury and death, which is involved in many complex factors such as oxidative stress, inflammation, and apoptosis. Disruption of the blood–brain barrier (BBB) was observed early after TBI, and the inflammatory response might be stimulated by the rapid release of molecular patterns, resulting in a significant local inflammatory response and intensifying brain damage (Navarrete et al., 2022). The neuroinflammatory response following TBI was known to be a key secondary injury factor, which can lead to neuronal damage (Corrigan et al., 2016). A strong inflammatory response was characterized by the recruitment and activation of immune cells, as well as the release of cytokines and chemokines. The immune status of the periphery directly impacted the inflammatory state of the neuropathology driven by the brain (Kempuraj et al., 2017). After a TBI event, aged mice showed different genetic characteristics of microglia and increased invasive T cells compared with young adult mice (Chen Z. et al., 2023). Therefore, the inhibition of neuroinflammation is a potential therapeutic target for TBI.

Recently, accumulating evidence indicated that gut microbiota and the brain were linked by the gut–brain axis. Gut microbiota are a symbiotic bacteria community that exists in the gut and may be closely related to gut–brain communication. Microbiota–gut–brain communication can theoretically occur through multiple pathways, such as endocrine, metabolic, and immune pathways. Accumulating evidence suggested that gut microbiota alterations might be involved in the progression and even deterioration of many diseases. Wang et al. analyzed the fecal bacterial communities in rats with mild traumatic brain injury (mTBI) at 12 different post-injury times, and the relative abundance of the bacterial communities had markedly varied at 2 h post-injury (Wang S. et al., 2023). Remarkably, gut microbiota dysbiosis was considered to be the main risk factor of TBI, which was likely to be alleviated by probiotic therapy. It was reported that antibiotic treatment could inhibit the gut microbiota of PGAM5(-/-) mice, partially alleviating post-TBI inflammatory response and motor dysfunction in mice, and *Akkermansia muciniphila* treatment inhibited neuroinflammation in the cerebral cortex after TBI (Chen Y. et al., 2023). Moreover, our previous study revealed that probiotic *Clostridium butyricum* treatment significantly improved neurological dysfunction, brain edema, and BBB impairment; reduced colonic IL-6 level; and protected the integrity of the intestinal barrier in the TBI mice (Li et al., 2018). Gut microbiota regulated the neuropathological changes through the microbiota–gut–brain axis. It was reported that quercetin could reverse the abnormalities of microbiota induced by repeated mild traumatic brain injury (rmTBI) and exerted a neuroprotective effect, which may be mediated by the regulation of the microbiota–gut–brain axis (Balasubramanian et al., 2023). The microbiota–gut–brain communication could be carried out by the immune system pathway. The leakage of microbiota or microbial metabolites from the intestine might lead to a systemic immune response, and pro-inflammatory cytokines might circulate to the brain, leading to subsequent brain inflammation. Th17 and Tregs were the most abundant lymphocytes in the lamina propria, and gut microbiota were crucial to the accumulation of these two cell types (Nishio et al., 2015). It was reported that gut microbiota could alter the ratio of pro-inflammatory Th17 to anti-inflammatory regulatory T cell (Treg), and the imbalance between Th17 and Treg cells was related to the development of TBI (Chen et al., 2022), indicating that gut microbiota might induce adaptive changes in immune response through the amplification of specific lymphocyte subtypes. With these lines of evidence, the immune pathway of the gut microbiota–gut–brain axis played an important role in TBI. Although the abnormal gut microbiota were closely related to the pathogenesis of TBI, the effects and mechanism of FMT against neuroinflammation of TBI are unclear.

In the present study, we explored the neuroprotective effects of FMT against neurological deficits, neuropathological changes, microglia activation, and neuroinflammation in the TBI mice by regulating the gut–brain axis. Meanwhile, gut microbiota composition and the population of Treg cells in the spleen were assessed. Moreover, we further confirmed that potential anti-inflammatory mechanisms were involved in the peripheral immune pathway, which would provide a potential strategy to alleviate TBI.

## 2 Materials and methods

### 2.1 Animals

Male C57BL/6J mice (2 months old) were purchased from the Hangzhou Ziyuan Experimental Animal Technology Co., Ltd., Hangzhou, China. Mice were raised under a constant temperature (22°C ± 1°C), stable humidity (50% ± 20%) with a 12-h light–dark cycle, as well as free access to food and water. All experiments were conducted in accordance with the guidelines of the Animal Experiment Ethics Committee of Wenzhou Medical University.

### 2.2 Induction of TBI

The TBI mouse model was induced by weight-drop impact head injury, as described previously (Li et al., 2016). Briefly, the mouse was anesthetized with 350 mg/kg of chloral hydrate and fixed on the operating table. A midline longitudinal incision was made on top of the mouse skull, the periosteum was peeled off, and the right parietal bone was exposed. A bone hole with a diameter of approximately 2 mm (located 2 mm next to the sagittal suture and 3 mm below the coronal suture) was drilled into the mouse. The weight (20 g) drops free fall at a height of 20 cm to impact the impact bar, and the end of the impact head is in vertical contact with the dura mater. After the impact, the impact device was immediately removed and bleeding was stopped with a gelatin sponge, and the scalp incision of the model mouse was sutured.

### 2.3 Experimental design

**Figure 1:**
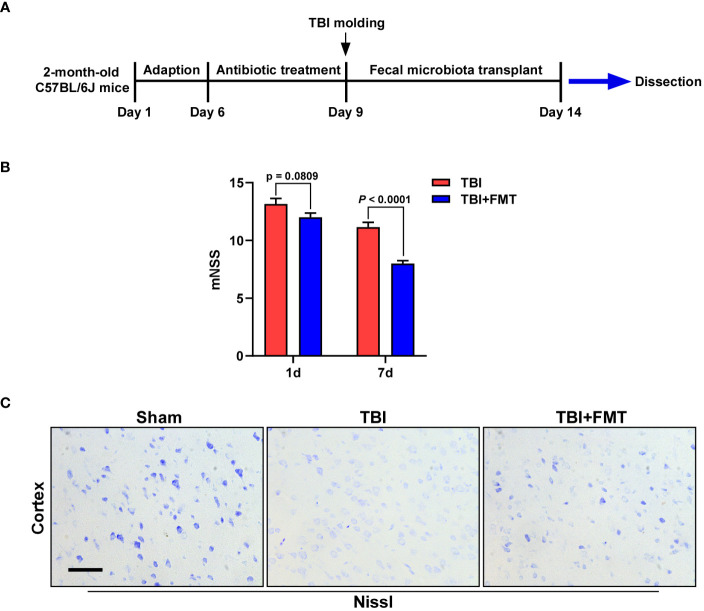
Fecal microbiota transplantation (FMT) treatment improved neurological deficit and neuronal damage in traumatic brain injury (TBI) mice. **(A)** The experimental schematic diagram. **(B)** The mNSS after FMT treatment (1 and 7 days) was assessed. Error bars meant SEM. *n* = 6–8 per group. **(C)** Representative images of Nissl staining. The neuronal damage of mice was determined by Nissl staining. Magnification: 400×, Scale bars: 50 μm.

TBI mice were randomly divided into two groups: (1) the TBI model group (TBI), which was subjected to weight-drop impact head injury, and (2) the TBI+FMT group (TBI+FMT), which was subjected to weight-drop impact head injury, and C57BL/6J mice intragastrically administered with 0.2 ml of fresh fecal solution once a day for 5 days. The stool (200 mg) from healthy C57BL/6J mice was collected daily and resuspended in 5 ml of sterile physiological saline and then passed through a 20-mm filter to remove large particulate. The filtrate was centrifuged at 3,000×*g* for 15 min and dissolved in physiological saline with a concentration of 400 mg/ml for transplantation. Before FMT treatment, mice were treated with 200 μl of combined antibiotics (vancomycin 100 mg/kg, ampicillin 200 mg/kg, and metronidazole 200 mg/kg) once a day for 3 days, as previously described with a modification (Wu et al., 2014; Zhou et al., 2017). The sham group (sham) underwent the same surgical procedure except for the weight drop. Mice of the sham and TBI groups were treated with the same dose of physiological saline. A diagram to describe these procedures is shown in **Figure 1A**.

### 2.4 Evaluation of neurological deficit

Neurological deficit was evaluated by an 18-point modified Neurological Severity Score (mNSS), as previously described (Zuo et al., 2021). mNSS was conducted 1 and 7 days after FMT treatment. The mNSS scoring criteria were as follows: (1) tail suspension test (3 points); (2) mouse was placed on the floor and its walking performance was evaluated (3 points); (3) sensory test (2 points); (4) balance beam test (6 points); and (5) loss of reflexes and abnormal movements (4 points). The mNSS score was a comprehensive evaluation of the motor, sensory, balance, reflex, and other neurological conditions of TBI mice, with a score range of 0–18 points; the larger the score, the more severe the neurological deficit.

### 2.5 Nissl staining

After the behavior test, the mice were anesthetized with 10% chloral hydrate and perfused with 4% paraformaldehyde. The brain tissues were quickly taken out, fixed in 4% paraformaldehyde at 4°C for 72 h, dehydrated with gradient ethanol for 48 h, embedded in paraffin, and cut into slices with a thickness of 5 μM. After baking at 65°C for 1.5 h, the slices were dewaxed with xylene for 15 min and then rehydrated with gradient ethanol and distilled water. The slices were stained with Nissl’s dye for 20 min and the color was separated with 70% alcohol. Then, the slices were dehydrated with gradient alcohol for 1 min each, fixed in xylene for 15 min, and sealed with neutral resin. The number of Nissl bodies was observed under a microscope, and representative photographs were taken.

### 2.6 Immunohistochemical analysis

The sections of the brain were immersed in citrate buffer, subjected to heat-induced antigen retrieval for 10 min, and cooled at room temperature. Then, the sections were placed in 3% H_2_O_2_ for 15 min, sealed with 5% bovine serum albumin (BSA) for 30 min, and incubated with primary antibodies (PSD-95, 1:200, Bioworld, MN, USA; synapsin I, 1:200, Bioworld, MN, USA; CD11b, 1:200, Bioworld, MN, USA) at 4°C overnight. The sections were incubated with horseradish peroxidase (HRP)-labeled secondary antibodies (Beyotime, Shanghai, China) at 37°C for 30 min, incubated with diaminobenzidine (DAB), and counterstained with hematoxylin. Then, the sections were differentiated with 1% hydrochloric acid for 3 s, dehydrated with gradient alcohol, cleared with xylene for 15 min, and sealed with neutral resin.

### 2.7 HE staining

The colon tissues of the mouse were removed, fixed in formaldehyde, embedded into paraffin, and then sliced. The slices were stained with hematoxylin for 8 min, differentiated with 1% hydrochloric acid alcohol for 3 s, and stained with eosin for 5 s. The slices were then dehydrated by gradient alcohol, fixed in xylene, and sealed with neutral resin.

### 2.8 Western blot

The brain tissue of the mouse was collected; added with RIPA lysate, PMSF, and phosphate protease inhibitor; and centrifuged at 12,000 rpm at 4°C for 20 min. The protein concentration of the sample was measured with bicinchoninic acid (BCA) solution and adjusted to the same concentration. The samples were subjected to SDS-PAGE electrophoresis and membrane transfer, sealed with 5% skim milk solution, and cut into corresponding bands. The bands were incubated with a dilution of primary antibody (PSD-95, 1:1,000, Bioworld, MN, USA; synapsin I, 1:1,000, Bioworld, MN, USA; CD11b, 1:1,000, Bioworld, MN, USA) overnight at 4°C, washed with PBST solution, and incubated with HRP-labeled secondary antibodies (1:5,000) at 37°C. Then, the bands were added with an ECL luminescent reagent, and the grayscale value of the band was analyzed using the ImageJ image processing system. β-Actin was used as the loading control.

### 2.9 Flow cytometry

The fresh spleen tissue from mice was obtained under sterile conditions and prepared as lymphocyte suspension (5 × 10^6^ cells/ml). CD4^+^CD25^+^FoxP3^+^ cells were stained and detected according to the manufacturer’s instruction of the mouse regulatory T-cell staining kit (Multisciences, Hangzhou, Zhejiang, China). Briefly, the cells were stained with fluorescent monoclonal antibodies of anti-CD4-FITC (Multisciences) and anti-CD25-APC (Multisciences), incubated in the dark at 2°C–8°C for 30 min. As for intracellular FoxP3 staining, the cells were treated with fixation/permeabilization solution (Multisciences) and subsequently incubated with the fluorescent antibody of anti-FoxP3-PE (Multisciences) at 2°C–8°C for 40 min. The acquisition and detection were performed on flow cytometry (FACSCalibur; Becton Dickinson, USA), and isotype-matched negative controls were used in the assay.

### 2.10 ELISA assay

The content of TNF-α in the brain tissue was determined by ELISA (No. RK00027, Westang Bio-Tech Co., Ltd., Shanghai, China) according to the manufacturer’s instructions. Brain tissue and lysate were mixed at a ratio of 1:9, added with PMSF, and homogenized with a homogenizer, and then the supernatant was taken as a sample for absorbance detection. The absorbance was measured by a microplate reader at the wavelength of 450 nm, and the sample concentration was calculated.

### 2.11 Gut microbiota analysis

The colon contents of mice were collected and stored at −80°C for 16S rRNA sequencing. The DNA of gut microbiota in each sample was extracted. The V3–V4 region was selected for PCR amplification, where the upstream primer was 338 F (ACTCCTACGGGGAGCAG) and the downstream primer was 806 R (GGACTACHVGGGTWTCTAAT). The purified PCR products were subjected to Qubit quantitative detection and were sequenced by the Illumina MiSeq platform (Illumina, San Diego, USA). The original sequencing was controlled by Trimmomatic software and spliced by FLASH software. The amplicon sequence variant (ASV) clustering was performed on sequences according to 100% similarity using DADA2 software, and single sequences and chimeras were removed in the clustering process. Each sequence was annotated species classification by using the Ribosomal Database Project (RDP) classifier. Finally, community diversity, community composition, and the species difference of the gut microbiota were analyzed, in which the LDA threshold was 4.0 in linear discriminant analysis effect size (LEfSe). The Simpson index and the principal coordinates analysis (PCoA) on the Bray–Curtis dissimilarity index were measured. The intestinal typing analysis was performed at the levels of phylum, family, and genus by clustering samples with similar dominant microbiota structures. Moreover, we identified the significant differences in the relative abundance of gut microbiota by the Wilcoxon rank sum tests based on the obtained community abundance data. Linear discriminant analysis (LDA) effect size (LEfSe) was applied to detect enriched taxa by the non-parametric Kruskal–Wallis (KW) rank-sum test.

### 2.12 Statistical analysis

The data were expressed as mean ± standard error of the mean (SEM) and analyzed using SPSS 26.0 software (SPSS, Chicago, IL, USA). The differences of the three groups of data were compared by one-way analysis of variance (ANOVA) and Tukey’s multiple comparisons test. The values from mNSS were analyzed by Student’s *t*-test. *P <*0.05 was regarded as a significant difference.

## 3 Results

### 3.1 FMT treatment improved neurological deficit and pathological changes in TBI mice

The neurological deficit of mice was detected by mNSS after treatment (1 and 7 days). As shown in **Figure 1B**, on the 1st day, the mNSS of the FMT treatment group was lower than that of the TBI group, but there was no significant difference between the two groups (*P* = 0.0809). On the 7th day, the mNSS in the FMT treatment group was significantly decreased than in the TBI group (*P* < 0.0001), suggesting that FMT could improve TBI-induced neurological deficit in mice. The neuronal damage of mice was determined by Nissl staining. As shown in **Figure 1C**, the number of Nissl bodies in the TBI group was lower than that in the sham group, while that in the FMT treatment group was higher than that in the TBI group, indicating that FMT treatment could improve neuronal damage of TBI.

### 3.2 FMT treatment improved synaptic damage in TBI mice

**Figure 2:**
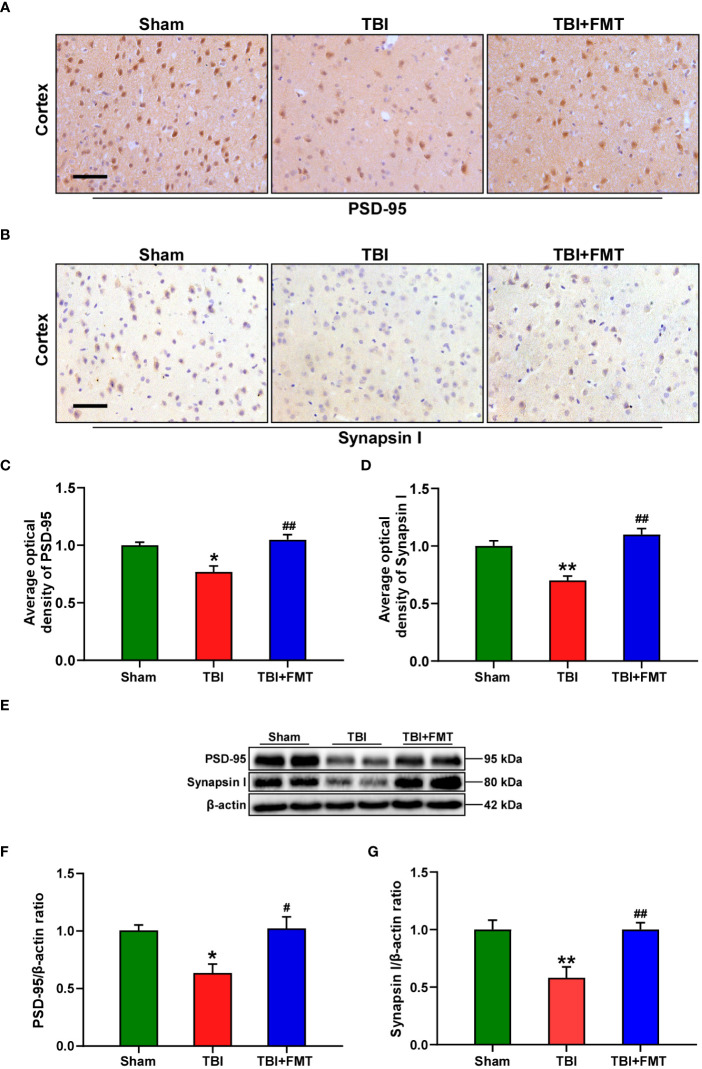
FMT treatment improved synaptic damage in TBI mice. **(A)** Representative immunohistochemistry images of PSD-95 in the cortex among sham, TBI, and TBI+FMT groups. **(B)** Representative immunohistochemistry images of synapsin I in the cortex among the three groups. Magnification: 400×, scale bars: 50 μm. **(C)** Comparison of the average optical density of PSD-95 among the three groups. **(D)** Comparison of the average optical density of synapsin I among the three groups. **(E)** Representative Western blot images of PSD-95 and synapsin I in the brain among the three groups. **(F)** Quantitative analysis of PSD-95; the ratio of PSD-95/β-actin in the sham group was used as a reference value. **(G)** Quantitative analysis of synapsin I: the ratio of synapsin I/β-actin in the sham group was used as a reference value. Error bars meant SEM; *n* = 4–6 per group. ^*^ *P* < 0.05, compared with the sham group; ^**^ *P* < 0.01, compared with the sham group; ^#^ *P* < 0.05, compared with the TBI group; ^##^ *P* < 0.01, compared with the TBI group.

The levels of synaptic plasticity protein PSD-95 and synapsin I were determined by immunohistochemistry and Western blot. The number of positive cells of PSD-95 and synapsin I in the TBI group was significantly lower than that in the sham group (PSD-95: *F* = 12.93, *P* < 0.05 vs. the sham group, **Figures 2A, C**; synapsin I: *F* = 21.06, *P* < 0.01 vs. the sham group, **Figures 2B, D**), while the number of positive cells of PSD-95 and synapsin I in the FMT treatment group was significantly higher than that in the TBI group (PSD-95: *P* < 0.01 vs. the TBI group, **Figures 2A, C**; synapsin I: *P* < 0.01, **Figures 2B, D**). To further verify these results, Western blot was performed. The level of PSD-95 in the TBI group was significantly reduced compared with the sham group (*F* = 6.70, *P* < 0.05 vs. the sham group, **Figures 2E, F**), while the level of PSD-95 in the FMT treatment group was significantly higher than that in the TBI group (*P* < 0.05 vs. the TBI group, **Figures 2E, F**). The level of synapsin I in the TBI group was significantly decreased compared with the sham group (*F* = 9.26, *P* < 0.01 vs. the sham group, **Figures 2E, G**), while the level of synapsin I in the FMT treatment group was significantly higher than that in the TBI group (*P* < 0.01 vs. the TBI group, **Figures 2E, G**). These results suggested that FMT treatment could improve the synaptic damage of TBI.

### 3.3 FMT treatment relieved neuroinflammation in TBI mice

**Figure 3:**
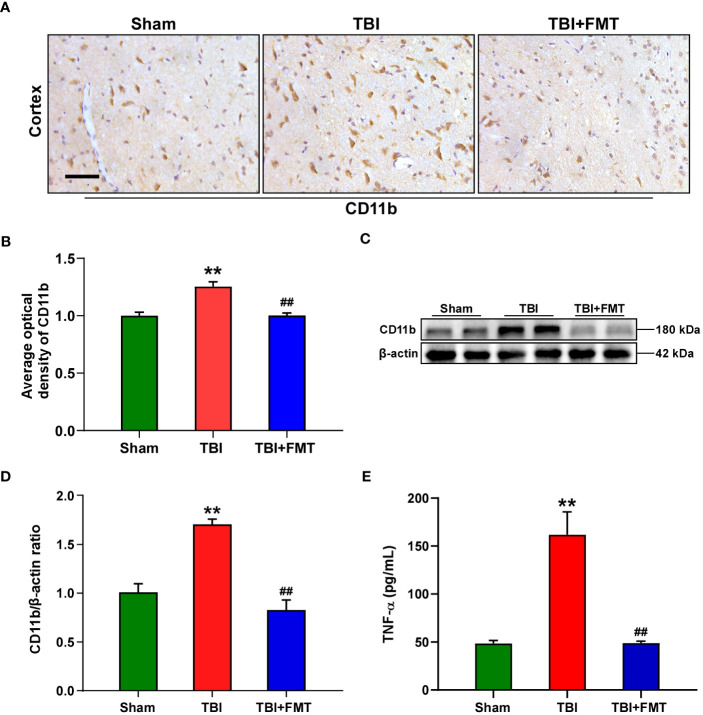
FMT treatment relieved neuroinflammation in TBI mice. **(A)** Representative immunohistochemistry images of CD11b in the cortex among the sham, TBI, and TBI+FMT groups. Magnification: 400×, scale bars: 50 μm. **(B)** Comparison of the average optical density of CD11b among the three groups. **(C)** Representative Western blot images of CD11b in the brain among the three groups. **(D)** Quantitative analysis of CD11b; the ratio of CD11b/β-actin in the sham group was used as a reference value. Error bars meant SEM; *n* = 4–6 per group. **(E)** Comparison of TNF-α level in the brain tissue among the three groups. ^**^ *P* < 0.01, compared with the sham group; ^##^ *P* < 0.01, compared with the TBI group.

As shown in **Figures 3A, B**, the number of CD11b-positive cells in the TBI group was significantly higher than that in the sham group (*F* = 20.88, *P* < 0.01 vs. the sham group), while the number of CD11b-positive cells in the FMT treatment group was significantly lower than that in the TBI group (*P* < 0.01 vs. the TBI group). To further verify these results, Western blot was performed. The level of CD11b in the TBI group was significantly higher than that in the sham group (*F* = 24.73, *P* < 0.01 vs. the Sham group, **Figures 3C, D**), while the level of CD11b in the FMT treatment group was significantly lower than that in the TBI group (*P* < 0.01 vs. the TBI group, **Figures 3C, D**). Reactivated microglia produced a series of inflammatory factors including TNF-α, which were determined by ELISA. As shown in **Figure 3E**, the level of TNF-α in the TBI group was higher than that in the sham group (*F* = 22.2, *P* < 0.01 vs. the sham group), while the level of TNF-α in the FMT treatment group was significantly lower than that in the TBI group (*P* < 0.01 vs. the TBI group). These results suggested that FMT treatment could inhibit the excessive microglia activation and suppress the neuroinflammatory reaction of TBI.

### 3.4 FMT treatment attenuated intestinal histopathologic changes in TBI mice

**Figure 4:**
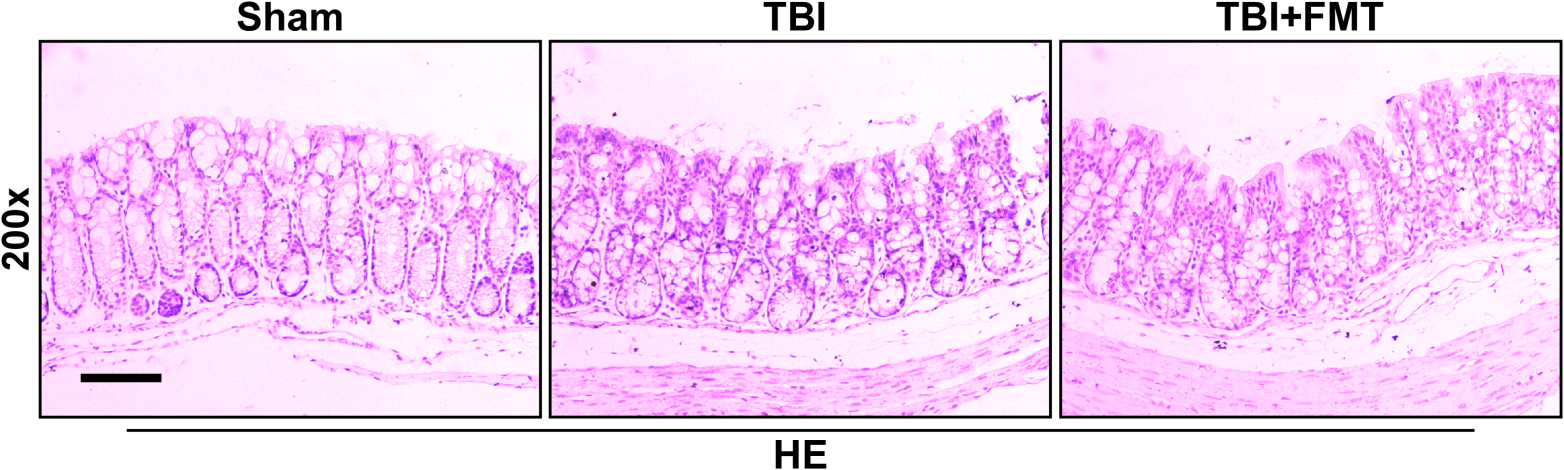
FMT treatment improved intestinal injury in TBI mice. The intestinal histopathologic changes in mice were detected by HE staining. Representative images of HE staining in the colon tissues among the sham, TBI, and TBI+FMT groups. Magnification: 200×, scale bar: 100 μm.

As shown in **Figure 4**, the intestinal histopathologic changes were detected by HE staining. The sham group showed that the villus was arranged neatly and the basement layer was intact. The TBI group showed cell damage and infiltration of inflammatory cells. However, FMT treatment significantly reduced cell damage and maintained the tissue structure, suggesting that FMT treatment might exhibit beneficial effects in attenuating TBI-induced intestinal injury.

### 3.5 FMT treatment improved gut microbial dysbiosis in TBI mice

**Figure 5:**
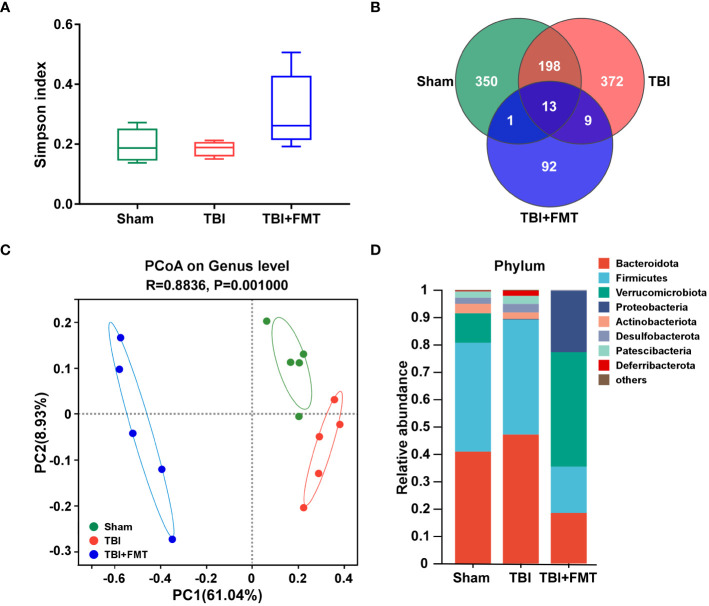
FMT treatment regulated gut microbial diversity and community composition in TBI mice. **(A)** Alpha diversity Simpson index analysis of gut microbiota. **(B)** The Venn diagram displayed the discrepancy and overlap of OTUs among the three groups. **(C)** PCoA showed variations of gut microbiota composition among the three groups, *P*-value was indicated, and each character represented a sample. **(D)** Comparison of different taxa at the phylum level among the three groups.

**Figure 6:**
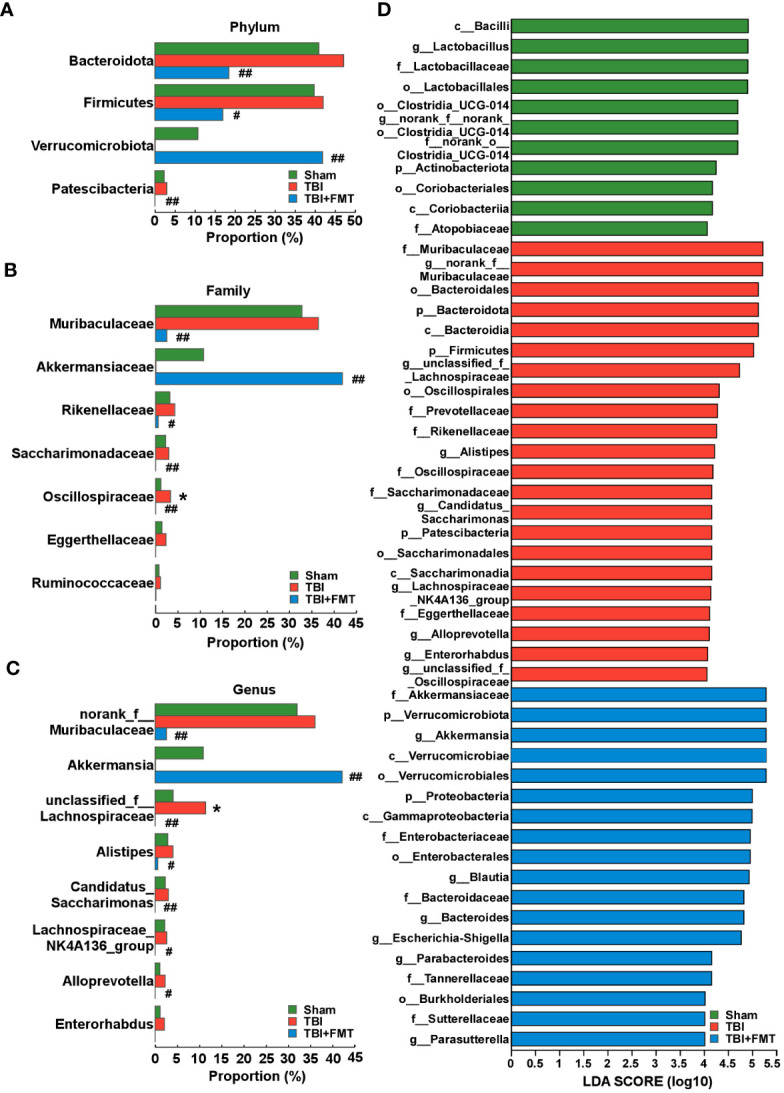
FMT treatment regulated the changes of differential microbiota in TBI mice. **(A)** Comparison of gut bacteria at the level of phylum among the sham, TBI, and TBI+FMT groups. **(B)** Comparison of gut bacteria at the level of family among the three groups. **(C)** Comparison of gut bacteria at the level of genus among the three groups. **(D)** LEfSe analysis revealed significant bacterial differences among the sham (green), TBI (red), and TBI+FMT (blue) groups. The LDA scores (log10) >4.0 and *P <*0.05 were listed. ^*^P < 0.05, compared with the sham group; ^#^ *P* < 0.05, compared with the TBI group; ^##^ *P* < 0.01, compared with the TBI group.

As shown in **Figure 5A**, there were no significant differences in the α-diversity Simpson index among the sham, TBI, and TBI+FMT groups (*P* > 0.05). As shown in **Figure 5B**, the Venn diagram displayed that there were 562, 592, and 115 specific OTUs in the three groups, respectively, and 13 operational taxonomic units (OTUs) were shared among these three groups. In addition, PCoA showed changes in microbial β-diversity, which was significantly different among the three groups (**Figure 5C**). As shown in **Figure 5D**, the alterations of community composition on the phylum level were observed. Then, we further evaluated the characteristic bacteria among the three groups. As shown in **Figure 6A**, at the phylum level, the relative abundance of *Verrucomicrobiota* in the TBI group was decreased compared with the sham group, while the abundance of *Verrucomicrobiota* in the TBI+FMT group was significantly increased than that in the TBI group (*P* < 0.01). As shown in **Figure 6B**, at the family level, the abundance of *Muribaculaceae* (*P* < 0.01), *Rikenellaceae* (*P* < 0.05), *Saccharimonadaceae* (*P* < 0.01), and *Oscillospiraceae* (*P* < 0.01) in the TBI+FMT group was significantly lower than that in the TBI group, and the relative abundance of *Akkermansiaceae* in the TBI+FMT group was significantly higher than that in the TBI group (*P* < 0.01). Moreover, as shown in **Figure 6C**, at the genus level, the abundance of *Alistipes* (*P* < 0.05), *Candidatus Saccharimonas* (*P* < 0.01), and *Alloprevotella* (*P* < 0.05) in the TBI+FMT group was significantly lower than that in the TBI group, and the relative abundance of *Akkermansia* (*P* < 0.01) in the TBI+FMT group was significantly higher than that in the TBI group. Furthermore, LEfSe analysis revealed significant taxonomic differences among the three groups. As shown in **Figure 6D**, *Muribaculaceae* and *Bacteroidales* in the TBI group were the dominant bacteria, while *Akkermansiaceae*, *Verrucomicrobiales*, and *Proteobacteria* were the dominant bacteria in the TBI+FMT group.

### 3.6 FMT treatment increased the population of Treg cells in TBI mice

**Figure 7:**
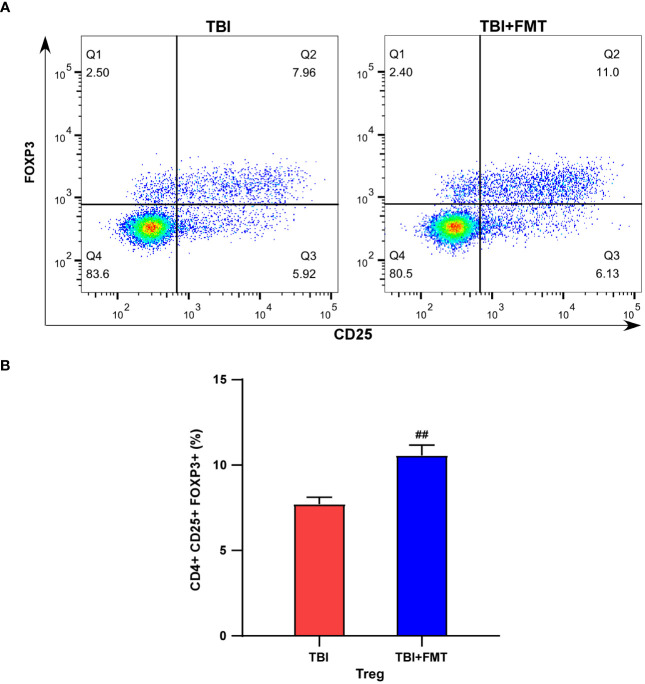
FMT treatment increased the population of Treg cells in TBI mice. **(A)** Representative flow cytometry images of Treg (CD4^+^CD25^+^FOXP3^+^) cells in the TBI and TBI+FMT groups. Blue indicated the number of rarer cells, and red indicated the number of denser cells; the Q2 quadrant represented Treg cells (CD4^+^CD25^+^FOXP3^+^). **(B)** The percentage of Treg (CD4^+^CD25^+^ FOXP3^+^) cells. ^##^ *P* < 0.01, compared with the TBI group.

As shown in **Figure 7**, we compared the CD4^+^CD25^+^FoxP3^+^ Treg cell population in the spleen between the TBI and FMT treatment groups. Our results showed that FMT treatment significantly increased the population of Treg cells in TBI mice (*P* < 0.01, **Figures 7A, B**), suggesting that FMT treatment could regulate peripheral immune cells of TBI.

## 4 Discussion

Recently, the anti-neuroinflammatory effect of gut microbiota was linked to the gut microbiota–gut–brain axis. In this study, we demonstrated that FMT treatment significantly relieved TBI-induced neurological deficits, neuronal damage, synaptic dysfunction, intestinal injury, neuroinflammation, and gut microbial disturbances in mice via regulating the gut microbiota–gut–brain axis and elucidated its underlying mechanism.

Neurological deficit was a hallmark of secondary brain injury after TBI and an important cause of limb disability in patients (Zhang S. et al., 2022). Du et al. reported that FMT (specific pathogen-free donor rats) could significantly restore general neurological function and improve learning ability in rats with TBI (Du et al., 2021). In this study, FMT improved the neurological deficit of TBI mice. Generally, neurological impairment after TBI was caused by its pathological changes, such as neuronal damage and synaptic plasticity dysfunction (Hernandez et al., 2018; Chen et al., 2020; McDaid et al., 2021; Wang et al., 2022). It was reported that neuronal cell death was observed in TBI mice (Xie et al., 2019), and the levels of synaptophysin and PSD-95 were reduced in TBI mice (Ritzel et al., 2020; Zhuang et al., 2023). Xiao et al. proved that FMT enhanced neuronal survival in the hippocampus in rats with chronic cerebral hypoperfusion (Xiao et al., 2022). In addition, FMT treatment remarkably restored the damage of dopaminergic neurons in the chronic rotenone-induced Parkinson’s disease mouse model (Zhao et al., 2021). Our previous studies have shown that FMT could increase synaptic plasticity by upregulating the levels of PSD-95 and synapsin I in the APP/PS1 mice (Sun et al., 2019). In this study, FMT reduced neuronal damage and increased the levels of PSD-95 and synapsin I in TBI mice, suggesting that FMT could improve the pathological changes of TBI.

Neuroinflammation was a key event after TBI, which exacerbated secondary brain injury and neurological deficits (Wu H. et al., 2021). Mounting studies indicated that there was a sustained neuroinflammatory process in TBI animal models (Radpour et al., 2022; Zhao et al., 2022). Kristina et al. reported that TBI induced the aggregation and activation of microglia and increased the mRNA expression of inflammation-related genes in mouse cortical tissue (Witcher et al., 2021). Our previous study showed that FMT downregulated the level of CD11b in APP/PS1 mice (Sun et al., 2019). Additionally, multitudinous pieces of evidence supported that FMT could downregulate the levels of the inflammatory factors IL-1β, IL-6, and TNF-α (Tang et al., 2021; Wen et al., 2022). Our results showed that FMT reduced the secretion of pro-inflammatory cytokines and inhibited the microglia reactivity, suggesting that FMT could relieve the neuroinflammation of TBI.

Intestinal injury was also a vital secondary pathological injury induced by TBI (Hanscom et al., 2021) and could be observed in TBI mice (You et al., 2022). Intestinal injury was often accompanied by disturbances in the gut microbiota, which existed in TBI mice (Treangen et al., 2018). It was reported that FMT enhanced intestinal barrier integrity in mice with spinal cord injury (Jing et al., 2021). In fact, several studies have shown a wide range of FMT intervention times. Kim et al. observed that a 5-day FMT from 5xFAD mice into C57BL/6 mice reduced adult hippocampal neurogenesis and brain-derived neurotrophic factor expression (Kim et al., 2021). Sun et al. reported that 7-day FMT treatment improved MPTP-induced motor dysfunction, increased striatal neurotransmitters, and improved dopaminergic neurons in mice (Sun et al., 2018). Wang et al. reported that 14-day FMT infusions improved MPTP-induced behavioral impairment and neuronal damage and reduced neuroinflammation in mice (Zhang T. et al., 2022). In this study, the 5-day FMT treatment could regulate the gut microbiota and inhibit the neuroinflammation of TBI mice. Taken together, these studies suggested the value of FMT in reconstructing gut microbiota, and different FMT intervention times might be due to differences in the diseases or animal models. In this study, *Bacteroidota* and *Muribaculaceae* were the dominant bacterial taxa in TBI mice, while FMT treatment could decrease the abundance of *Bacteroidota* and *Muribaculaceae*. Additionally, the study has shown the increased abundance of *Muribaculum*, the genus type of the family *Muribaculaceae*, order *Bacteroidales*, and phylum *Bacteroidota* in TBI mice (Chen Y. et al., 2023). In addition, our results showed that FMT treatment decreased the relative abundances of *Patescibacteria*, *Saccharimonadaceae*, *Rikenellaceae*, *Alistipes*, *Ruminococcaceae*, and *Eggerthellaceae* in TBI mice, which was consistent with previous studies. For instance, it was reported that the abundance of *Patescibacteria*, *Saccharimonadales*, and *Saccharimonadia* was increased in patients with Alzheimer’s disease and mild cognitive impairment (Zhu et al., 2022), and *Patescibacteria* was enriched in mice with neurobehavioral impairments (Diao et al., 2021). Furthermore, *Rikenellaceae* was the main dominant bacteria in mice after 28 days of TBI injury (Zheng et al., 2022), and the abundance of *Alistipes* increased in patients with chronic TBI (Urban et al., 2020). Moreover, the levels of *Rikenellaceae* and *Alistipes* were increased in rats with anxietylike behavior, accompanied by the activation of neuroinflammatory microglia and the increase of IL-1β expression in the hippocampus (Wu L. et al., 2021). Furthermore, the abundance of *Ruminococcaceae* was higher in moderate or severe TBI patients compared to controls (Urban et al., 2020). The relative abundance of *Ruminococcaceae* and *Eggerthellaceae* was significantly increased in high-status negative cognitive processing bias adults and cognitive dysfunction mice (Han et al., 2020; Xu et al., 2023). On the contrary, in this study, FMT increased the relative abundance of *Verrucomicrobiota*, *Akkermansiaceae*, and *Akkermansia* in TBI mice. *Akkermansia*, belonging to the family *Akkermansiaceae* of the phylum *Verrucomicrobiota*, exerted anti-inflammatory effects (Qu et al., 2021). The study has shown that the relative abundance of *A. muciniphila* was negatively correlated with intestinal injury and neurological dysfunction, as well as the levels of TNF-α and IL-1β in cortical tissue after TBI, and *A. muciniphila* treatment improved intestinal injury, neurological dysfunction, and neuroinflammation in the cerebral cortex of TBI mice (Chen Y. et al., 2023). To sum up, the enrichment of inflammation-related bacteria, such as *Rikenellaceae* and *Alistipes*, and the reduction of anti-inflammatory-related bacteria *Akkermansia* might participate in the process of TBI, and these changes in the relative abundance of bacteria could be reversed by FMT, which contributed to the explanation of the specific therapeutic mechanism of FMT in TBI.

The gut microbiota–gut–brain axis achieved functional interactions between the gut and brain through many pathways, such as the immune pathway (Mayer et al., 2022), which was closely related to gut microbiota (Yu et al., 2022). The alterations of gut microbiota stimulated peripheral immune cells and regulated neuroinflammation in the brain. The saponin treatment mediated the regulation of gut microbiota, significantly alleviated neurological impairment, and downregulated the ratio of Th17/Treg in the spleen of mice with autoimmune encephalomyelitis (EAE) (Wang J. et al., 2023). FMT from patients with rheumatoid arthritis induced a T-cell increase in the spleen, leading to depression-like behaviors in mice (Pu et al., 2022). Yan F. et al. confirmed that a positive correlation was observed between *Akkermaniacaeae* and peripheral anti-inflammatory macrophage CD8^+^ T cells in patients with hepatitis B virus associated hepatocellular carcinoma (HBV-HCC) (Yan et al., 2023). Additionally, the abundance of *Bacteroides* was positively correlated with the plasma level of TNF-α (Lin et al., 2019). Significantly, Treg cells, as regulatory T cells, acted on other immune cells through cell contact or secretion of anti-inflammatory cytokines, played an important role in adaptive immunity and maintaining immune homeostasis (Ohkura and Sakaguchi, 2020; Scheinecker et al., 2020), and had a close relationship to TBI. The infiltrating Tregs were observed in the brain of mice after 3 days of TBI and further increased after 7 days of TBI, while the absence of Tregs significantly reduced the protective effect of treatment measures on TBI mice, which indicated that regulating Treg-mediated peripheral immunity contributed to the improvement of TBI (Xie et al., 2022). In this study, FMT treatment significantly increased the percentage of Treg cells in the TBI. These results indicated that gut microbiota might interact with peripheral immune cells, affecting the activation of microglia, and then participate in regulating neuroinflammation of TBI, while the mechanism of gut microbiota–gut–brain communication is necessary to be further explored. Our hypothesis regarding this anti-neuroinflammatory mechanism of FMT is presented in **Figure S1**. In conclusion, our findings elucidated that FMT treatment effectively suppressed TBI-induced neuroinflammation in mice via regulating the gut microbiota–gut–brain axis, and its mechanism was involved in the regulation of peripheral immune cells, which provided insights for clinical strategies to alleviate TBI.

## Data availability statement

The datasets presented in this study can be found in online repositories. The names of the repository/repositories and accession number(s) can be found below: https://www.ncbi.nlm.nih.gov/, bioproject/PRJNA992248.

## Ethics statement

The animal study was approved by Animal Experiment Ethics Committee of Wenzhou Medical University. The study was conducted in accordance with the local legislation and institutional requirements.

## Author contributions

XH: Investigation, Funding acquisition, Methodology, Validation, Writing – original draft. HJ: Investigation, Methodology, Writing – original draft. SY: Investigation, Methodology, Writing – original draft. TY: Investigation, Formal Analysis, Methodology, Writing – original draft. ZC: Investigation, Formal Analysis, Methodology, Writing – original draft. YK: Formal Analysis, Investigation, Methodology, Writing – original draft. JL: Project administration, Supervision, Methodology, Writing – original draft. KX: Project administration, Investigation, Methodology, Writing – original draft. JS: Project administration, Supervision, Formal Analysis, Methodology, Software, Writing – original draft.

## Supplementary material

Supplementary Figure 1 Schematic diagram of the mechanism. FMT exerted beneficial effects against neuroinflammation of TBI via gut microbiota-gut-brain axis.
