# Supplementary figures and images for: Fecal microbiota transplantation inhibited neuroinflammation of traumatic brain injury in mice via regulating the gut–brain axis
Source: Front Cell Infect Microbiol. 2023 Sep 7;13:1254610. doi: 10.3389/fcimb.2023.1254610 (PMC10513427; doi:10.3389/fcimb.2023.1254610)

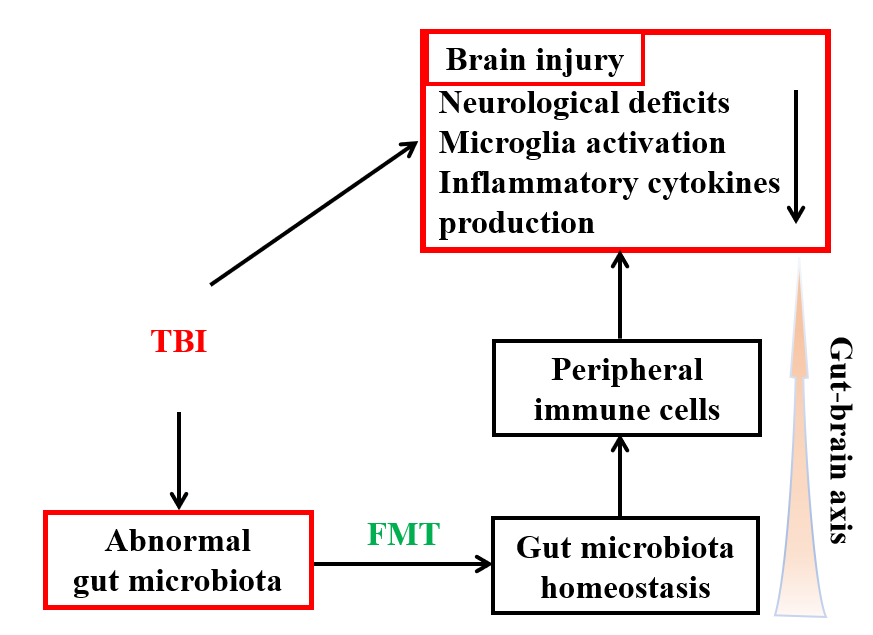

Supplement: Supplementary Figure 1 — Schematic diagram of the mechanism. FMT exerted beneficial effects against neuroinflammation of TBI via gut microbiota-gut-brain axis. [file Image_1.tif]
